# Mutation Distribution in the NSP4 Protein in Rotaviruses Isolated from Mexican Children with Moderate to Severe Gastroenteritis

**DOI:** 10.3390/v5030792

**Published:** 2013-03-11

**Authors:** Guadalupe González-Ochoa, Griselda E. Menchaca, Carlos E. Hernández, Cristina Rodríguez, Reyes S. Tamez, Juan F. Contreras

**Affiliations:** Facultad de Ciencias Biológicas, Universidad Autónoma de Nuevo León, Av. Universidad S/N Ciudad Universitaria, San Nicolás de los Garza, Nuevo León, CP. 66451, México

**Keywords:** rotavirus, severe gastroenteritis, NSP4

## Abstract

The NSP4 protein is a multifunctional protein that plays a role in the morphogenesis and pathogenesis of the rotavirus. Although NSP4 is considered an enterotoxin, the relationship between gastroenteritis severity and amino acid variations in NSP4 of the human rotavirus remains unclear. In this study, we analyzed the sequence diversity of NSP4 and the severity of gastroenteritis of children with moderate to severe gastroenteritis. The rotavirus-infected children were hospitalized before the rotavirus vaccine program in Mexico. All children had diarrhea within 1−4 days, 44 (88%) were vomiting and 35 (70%) had fevers. The severity analysis showed that 13 (26%) cases had mild gastroenteritis, 23 (46%) moderate gastroenteritis and 14 (28%) severe. NSP4 phylogenetic analysis showed three clusters within the genotype E1. Sequence analysis revealed similar mutations inside each cluster, and an uncommon variation in residue 144 was found in five of the Mexican NSP4 sequences. Most of the amino acid variations were located in the VP4 and VP6 binding site domains, with no relationship to different grades of gastroenteritis. This finding indicates that severe gastroenteritis caused by the rotavirus appears to be related to diverse viral or cellular factors instead of NSP4 activity as a unique pathogenic factor.

## 1. Introduction

Rotaviruses cause gastroenteritis in almost all mammals and some birds [1]. Group A, B and C rotaviruses are known to infect humans and animals; however, group A is responsible for gastroenteritis in children less than five years old [2]. The common symptoms of this disease are diarrhea, fever and vomiting [3]. Dehydration is a consequence of severe diarrhea that may cause infant death [2,3]. Statistics reveal that rotaviruses cause approximately 453,000 child deaths per year worldwide, with the highest mortality rates primarily in developing countries [4]. The rotavirus pathogenesis is related to the non-structural protein 4 (NSP4), which is a known enterotoxin [5]. NSP4 induces an intracellular calcium imbalance, resulting in membrane instability and loss of water; the same effect would be present by phospholipase C-mediated inositol 1,4,5-trisphosphate production when NSP4 interacts with non-infected cells [2,6,7,8].

NSP4 is a glycosylated protein of 175 amino acids and a molecular mass of 28 kDa in its mature form. This protein is characterized by three hydrophobic domains named H1 (residues 7−21), H2 (29−47) and H3 (67−85) and a coiled *α-*helical domain (95−137) [9]. The NSP4 amino-terminal region (1−44) is located in the lumen of the endoplasmic reticulum, whereas its carboxy-terminal region (45–175) is in the cytoplasm and interacts with different proteins including VP6 and VP4 during rotavirus morphogenesis [10,11,12]. NSP4 also interacts with some cellular proteins and extracellular matrix proteins [13,14,15].

On the other hand, the NSP4 sequence analysis has revealed at least 14 genotypes named E1−E14 (for Enterotoxin). The human rotavirus genotypes are E1 (Wa-like), E2 (Kun-like) and E3 (AU-1), previously known as genotypes B, A and C, respectively [16]. Information related to rotavirus infection and the role of NSP4 pathogenesis in humans has not been described in detail. Some reports indicate that changes in the sequence of NSP4, VP4 and VP7 are related with asymptomatic strains isolated from humans [17,18], but amino acids variation in NSP4 has not always been associated with asymptomatic infections [19,20,21]. In this study, we analyzed NSP4 of human rotavirus strains in Mexican children with different grades of gastroenteritis to determine the genotype, distribution and frequency of mutations in NSP4.

## 2. Results and Discussion

### 2.1. Rotavirus Positive Samples and Gastroenteritis Severity

A total of 123 diarrheic feces collected from October 2004 to March 2005 from hospitalized children with gastroenteritis in Monterrey, Nuevo Leon, México, were analyzed to detect rotavirus. Of all the analyzed samples, sixty-six (53.7%) were positive for the presence of a rotavirus. This is a high percentage, because usually rotavirus infection is associated with 25−39% of hospitalizations for acute gastroenteritis [22]. To further analyze the gastroenteritis severity, 16 of the 66 rotavirus positive samples were discarded due to incomplete data about the infected children or their symptoms. The remaining 50 gastroenteritis cases were considered in this study and the signs and symptoms of the rotavirus gastroenteritis were examined. Twenty-eight (56%) cases were male children and 22 (44%) were female children. Most of the rotavirus gastroenteritis cases (86%) were related to children under two years old, this is in agreement with previous reports on rotavirus infection [23,24,25]. All the infected children included in this study had diarrhea within 1 to 4 days, 44 (88%) of them were vomiting and 35 (70%) had a fever. The results of the rotavirus gastroenteritis severity showed that 13 (26%) cases had a score ≤ 10, whereas 23 (46%) infections showed a score ≥ 11 and 14 (28%) cases had a score ≥ 15. Those scores were grouped as mild, moderate and severe gastroenteritis, respectively (Table 1). Although some studies describe breastfeeding as an important factor to avoid severe gastroenteritis, we did not find a relationship between breastfeeding and the rotavirus gastroenteritis severity [26,27,28]. This type of analysis of rotavirus regarding the prevalence and severity of the gastroenteritis may represent a basis to compare the epidemic seasons of the rotavirus before and after the introduction of the Rotarix® vaccine (GSK) [29]. In this aspect, some studies have shown that this vaccine can diminish the cases of hospitalization by the rotavirus from 40 to 60% [30,31,32]. Additionally, studies in Latin America have shown the efficiency of the vaccine to decrease the severity of the rotavirus gastroenteritis [33,34].

### 2.2. The NSP4 Genotype

NSP4 is a main factor of rotavirus pathogenesis [35,36]. Therefore, in this study we have focused on the analysis of the NSP4 genotype and its sequence relationship with the rotavirus gastroenteritis severity. To amplify and identify the NSP4 genotype in all the rotavirus positive samples we used a combination of different primers that have been previously described [20,21,37]. This was a useful strategy that identified 61 (92.4%) of the samples as NSP4 genotype E1. The predominant NSP4 genotype E1 identified in the studied area is commonly reported; some studies identified the E2 genotype as the second common genotype [38,39,40,41]. Furthermore, others reported non-common NSP4 genotypes E3, E5, E6 and E13 in human rotavirus strains in Thailand, Brazil, Bangladesh and Kenya [42,43,44,45,46].

### 2.3. The NSP4 Sequence Analysis

**Figure 1:**
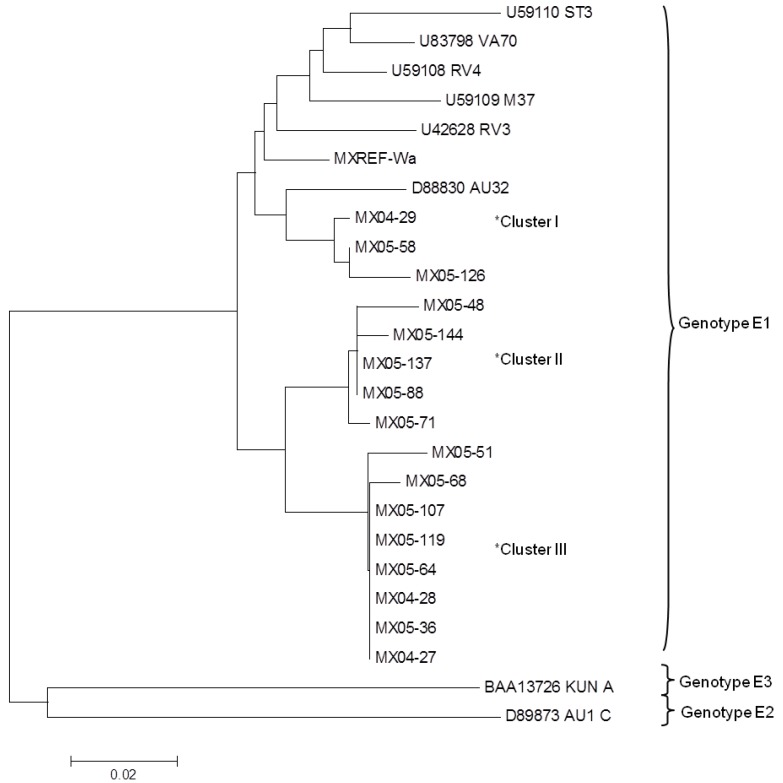
Phylogenetic analysis of the deduced amino acid NSP4 sequences reported in this study and other NSP4 genotypes previously reported in the NCBI. The phylogenetic tree was constructed based on the neighbor-joining method. The bootstrap consensus tree inferred from 500 replicates is taken to represent the evolutionary history of the taxa analyzed. The evolutionary distances were computed using the p-distance method and are in the units of the number of amino acid differences per site. Evolutionary analyses were conducted in MEGA5 package [54]. Accession number of the sequences reported in this study in the GenBank: MX04-28: JX458969, MX04-29: JX458970, MX04-27:JX458971, MX05-51:JX458972, MX05-144: JX458973, MX05-58: JX458974, MX05-68: JX458975, MX05-71:JX458976, MX05-88:JX458977, MX05-119:JX458978, MX05-107: JX458982, MX05-126: JX458983, MX05-137: JX458984.

According to the gastroenteritis severity analysis (Table 1), the rotavirus positive samples were classified as mild (26%), moderate (46%) and severe (28%). Based on these results a stratified random sampling was done to select representative samples to the NSP4 sequence analysis, this selection included 4(31%) of 13 samples of mild cases, 7 (30%) of 23 cases of moderate and 5 (36%) of 14 severe cases. The analysis of the deduced amino acid sequences of NSP4 reported in this study showed three clusters inside the same genotype E1 (Figure 1). The NSP4 sequences MX04-29, MX05-58, MX05-126 reported here grouped in the cluster I; the samples MX05-48, MX05-71, MX05-88, MX05-137, and MX05-144 were in the cluster II and the samples MX04-27, MX04-28, MX05-36, MX05-51, MX05-64, MX05-68, MX05-107 and MX05-119 in the cluster III (Table 3). Previous reports have shown the presence of at least two clusters within this genotype, and in some of them the clusters were related to the location or to the isolation date of the rotavirus strains, in this study we did not observed such relation [41,47,48]. The NSP4 amino acids variations showed in the cluster I (amino acids AK in position 136-137 respectively) were related with rotavirus strains reported in Italy, China, Spain, United States of America (USA) and Russia (Accession number ACF77154, AFU36983, ADU55685, ADO78536 and ACY01369). The sequences in the cluster II share the same amino acid variations in the positions 141-145 with sequences from China, Russia, Thailand and USA (AAOO6852, ACQ99541, AFQ20926, ADO78564) and the cluster III shared common amino acid variations in aa 141,142, 144 and 145 with the strain Vanderbilt isolated in USA (AEB80046). Further analyses on NSP4 were performed using the amino acid frequency in each specific position in the protein. The analysis of 349 NSP4 sequences from the GenBank database showed that this protein is highly conserved in some specific domains (Table 2). These conserved regions include the glycosylation sites (aa 8 and 18), the hydrophobic region H1, H2 and H3 (aa 7−21, 29−47 and 67−85), the transmembrane domain (aa 22−44) and the coiled *α-*helical domain (aa 97−137) where the frequency of the consensus amino acid in a specific position was 97.6 to 100%; however, the H3 domain showed an amino acid frequency of 88.9% for I72 and 87.4% for I76 ( Table 2). Conversely, punctual variations in the NSP4 sequence fell in the VP4 binding site domain where the lowest amino acid frequency was at position 141 with a valine present for 58% of the studied sequences, and also the VP6 binding site where a serine at position 169 had a frequency of 56% ( Table 2). Most of the amino acids variations in the NSP4 sequences reported were positioned in the carboxyl terminal region (Table 3). However, the samples MX04-29, MX05-58 and MX05-126 showed punctual variations in conserved amino acids 111, 136 and 137 (85.7−92.8%), respectively (Table 3). In addition, five of the NSP4 sequences reported in this study had an uncommon amino acid change at position 144, where a methionine was replaced by a valine. Usually methionine is present in this position in 97.9% of all the 349 NSP4 sequences analyzed (Figure 1), and thus this amino acid variation is unique in our sequences. Moreover, we did not find valine in this position in any other NSP4 genotype E1 sequence in the NCBI database. The replacement of a methionine may be not significant when it is replaced by another hydrophobic amino acid such as valine, because both amino acids can play a role in binding or recognition of hydrophobic ligands such as lipids. However, the sulfur atom in methionine can be involved in binding metals [49] and NSP4 presents a metal binding domain between residues 114 and 135 [50,51]; nevertheless, further analysis is required to explain the importance of the mutations in such conserved amino acid position within NSP4.

**Table 1:** Characteristics and symptoms of the children infected with the rotavirus and the analysis of the rotavirus severity score from slight to severe according to the Ruuska score [52].

Gastroenteritis severity (Ruuska score)	Gastroenteritis severity	Incidence	Breastfeeding	Sex		Age* (months)	diarrhea episodes* /24 h	Days with diarrhea*	Vomiting episodes* / 24hrs	Days of vomiting*
				M	F					
≤ 10	Mild	13 (26%)	61.5%	6	7	8	6.1	2.4	2.6	1.0
≥ 11	Moderate	23 (46%)	82.3%	15	8	12	8.1	3.1	4.8	2.7
≥ 15	Severe	14 (28%)	71.4%	7	7	14	10.4	4.3	9.3	3.6

* Average Data

**Table 2:** Consensus NSP4 sequence and the frequency of each amino acid in a specific position from residues 1 to 175. The analysis was obtained from 349 NSP4 sequences from the GenBank and is complemented with the description of some of the NSP4 domains. Abbreviations: **GS:** Glycosylation site, **H**: Hydrophobic domain.

																						Transmembrane site (22−44)			
								GS1	H1 (aa 7−21)									GS2							
	
Consensus seq	M	D	K	L	A	D	L	N	Y	T	L	S	V	I	T	L	M	N	D	T	L	H	S	I	I
Frequency %	100	98.5	99.7	98	97.6	99.5	100	99.7	100	99.4	99.4	92.7	99.7	97.9	98.2	99	100	99.4	97.9	98.8	100	98.8	98.8	100	98.8
aa position	26	27	28	29	30	31	32	33	34	35	36	37	38	39	40	41	42	43	44	45	46	47	48	49	50
				H2 (29−47)																					
	Transmembrane domain (22−44)																								
Consensus seq.	Q	D	P	G	M	A	Y	F	P	Y	I	A	S	V	L	T	V	L	F	T	L	H	K	A	S
Frequency %	97.6	99.7	100	100	96.4	100	100	100	97.8	100	98.8	100	99.7	99.4	100	100	994	99.4	99.7	98.4	100	99.4	99.4	100	99.4
aa position	51	52	53	54	55	56	57	58	59	60	61	62	63	64	65	66	67	68	69	70	71	72	73	74	75
																	H3 ( aa 67-85)								
Consensus seq.	I	P	T	M	K	I	A	L	K	T	S	K	C	S	Y	K	V	I	K	Y	C	I	V	T	I
Frequency %	99.4	99.4	99.1	99	99.7	99.4	100	99.4	96.6	99.4	100	98.8	99.1	100	99.7	100	99.4	97.9	99.1	98.5	98.8	88.9	99.1	99.4	95.5
aa position	76	77	78	79	80	81	82	83	84	85	86	87	88	89	90	91	92	93	94	95	96	97	98	99	100
	H3 (67−85)																			Alpha coiled Domain (95−137)					
Consensus seq.	I	N	T	L	L	K	L	A	G	Y	K	E	Q	V	T	T	K	D	E	I	E	Q	Q	M	D
Frequency %	87.4	99.7	98.5	100	99.1	98.8	98.2	98.8	100	99.4	98.5	100	99.1	96.7	98.8	98	99.1	98.5	98.8	99.7	100	98.2	99.7	99.7	99.1
aa position	101	102	103	104	105	106	107	108	109	110	111	112	113	114	115	116	117	118	119	120	121	122	123	124	125
	Alpha coiled Domain (95−137)																								
												VP4 Binding site (112−148)													
														Enterotoxin (114-135)											
Consensus seq.	R	I	V	K	E	M	R	R	Q	L	E	M	I	D	K	L	T	T	R	E	I	E	Q	V	E
Frequency %	99.7	98.8	99.1	99	99.7	99.4	99.7	100	98.5	99.7	93.4	99.4	99.7	99.1	99.1	100	100	98.8	99.1	99.7	99.7	99.7	99.7	99.4	100
aa position	126	127	128	129	130	131	132	133	134	135	136	137	138	139	140	141	142	143	144	145	146	147	148	149	150
	Alpha coiled Domain (95−137)																								
	VP4 Binding site (112−148)																								
	Enterotoxin (114−135)																								
Consensus seq.	L	L	K	R	I	H	D	N	L	I	T	R	P	V	D	V	I	D	M	S	K	E	F	N	Q
Frequency %	99.4	100	99.7	99	99.7	92.8	99.1	78.6	100	90.7	93.4	87.1	78.4	88	89.8	58	76	99.4	97.9	64.3	99.1	99.1	97.3	99.1	100
aa position	151	152	153	154	155	156	157	158	159	160	161	162	163	164	165	166	167	168	169	170	171	172	173	174	175
																	VP6 Binding site (167−175)								
Consensus seq.	K	N	I	K	T	L	D	E	W	E	S	G	K	N	P	Y	E	P	S	E	V	T	A	S	M
Frequency %	98.8	99.7	94	93	100	99.7	98.2	99.1	100	95.8	77	99.7	97.6	99.1	100	100	99.1	100	56	99.1	99.4	99.7	100	98.8	100

**Table 3:** Sequence analysis of the amino acid variations in the NSP4 protein from rotavirus strains reported in this study.

NSP4 Sequence	Severity scores	Cluster	Amino acid variability and distribution																	
			H3 *			TD	ACD *	E/VP4 *	VP4 *						VP6 *					
			76	77	85	94	111	132	136	137	141	142	144	145	161	169	172	173	174	175
MX04-29	16	I					N		A	K										
MX05-58	14	I					D		A	K										
MX05-126	16	I		V			D		A	K										
MX05-48	8	II	V		H						T			T	N	I				
MX05-144	13	II	V								T			T	N	I				
MX05-137	15	II	V								T			T	N	I				
MX05-88	16	II	V								T			T	N	I				
MX05-71	0	II	V								T			T	N	M				
MX04-27	11	III									S	V	V	T		I				
MX04-28	14	III									S	V	V	T		I				
MX05-36	8	III									S	V	V	T		I				
MX05-51	14	III						N			S	V	V	T		I	I			
MX05-64	12	III									S	V	V	T		I				
MX05-68	8	III				G					S	V	V	T		I				
MX05-107	14	III									S	V	V	T		I				
MX05-119	15	III									S	V	V	T	N	I				
**Consensus amino acids**			**I**	**N**	**Y**	**E**	**E**	**D**	**T**	**R**	**V**	**I**	**M**	**S**	**S**	**S**	**T**	**A**	**S**	**M**
**Frequency %**			**86**	**99.7**	**99.4**	**99.1**	**89.4**	**99.4**	**92.8**	**85.7**	**56.2**	**75.9**	**99.4**	**61**	**76**	**56**	**100**	**100**	**99**	**100**

* **H3: **hydrophobic region 3; **TD:** Tetramerization Domain; ACD: Alpha Coiled Coil Domain **E: **Enterotoxin Domain; **VP4: **VP4 Binding site domain; **VP6: **VP6 binding site domain

## 3. Experimental Section

### 3.1. Samples Recollection and Gastroenteritis Severity Score

A total of 123 stool samples were collected from hospitalized children with gastroenteritis in Monterrey, Nuevo León, México, from October 2004 to March 2005. The inclusion criteria were the age of the child, up to five years old, and the hospitalization for nonbacterial gastroenteritis. Diarrhea, vomiting and fever were used as registered symptoms to calculate the gastroenteritis severity, and formed the basis of the Ruuska score [52].

### 3.2. Extraction of Rotavirus RNA.

The feces samples were used to isolate, purify and detect the rotavirus RNA genome by TRI Reagent® (Molecular Research Center, Cincinnati, OH) as suggested the by supplier. The viral RNA was loaded onto a 10% polyacrylamide gel under native conditions, and then stained by a silver-staining procedure. A sample was considered positive to the rotavirus when a characteristic double-stranded RNA genome was observed in the gel [53].

### 3.3. NSP4 Genotype Identification and Sequence

RNA positive samples for the rotavirus were retro transcribed and amplified by PCR to isolate the NSP4 gene using the primers Beg16-End722 or NSP41F-NSP42R [20,21]; although in several experiments, combinations of both primer pairs were required to achieve amplification. NSP4 genotype identification was performed by a multiplex-seminested PCR, with 10END722 or NSP42R as the external primers and Wa, Kun or RRV as the internal primers, which corresponds to genotypes E1, E2 and E3, respectively [37]. Samples of the amplified NSP4 gene were cloned using the pGEM-T vector (Promega Inc, Madison, WI) according to the manufacturer´s instructions. The plasmid with the NSP4 insert was purified by the Wizard SV Minipreps kit (Promega Inc, Madison, WI) and sequenced by the dideoxynucleotide chain termination method, using an ABI Prism Big Dye Terminator Cycle Sequencing Ready Reaction kit (PE Applied Biosystems, Whashington, DC). The DNA sequence was confirmed by sequencing both DNA strands of each of the different clones using the pUCM13 sense and antisense standard primers. The resulting sequences were analyzed with MEGA 5.0 and compared with other sequences reported in the GenBank data base; the phylogenetic tree was determined by the Neighbor joining method [54]. The GenBank accession numbers of NSP4 sequences used in sequence analysis were AB361285, AB008233, AB008237, AB213391, AB326290, AB326294, AB326963, AB361282, AF170830, AF260930, AY159640, AY159642, AY353740, AY353800, DQ909069, DQ909070, EF033202, EF033203, EF672575, EU679377, EU679382, U42628, U83798, AAO06852, AAT48079, AB008229 - AB008231, AB008234-AB008236, AB008238-AB008245, AB008247-AB008257, AB008259 - AB008263, AB022772, AB043026, AB043069-AB043078, AB196491, AB196492, AB196958, AB196959, AB211987-AB213392, AB232699, AB269688, AB303218, AB326286-AB326289, AB326293, AB326295, AB326297, AB326334, AB326336, AB326337, AB326347, AB326348, AB326962, AB326966, AB326969, AB326971, AB361276, AB361281, AB361284, AB361286-AB361288, ABK62862, ABU49806, ACF77153, ACF77154, ACJ54826, ACJ66758, ACJ66769, ACL80635, ACL80638, ACQ99541, ACY01369, ACY01381, ACZ51671, ADA70484, ADK46705, ADK46715, ADO78533, ADO78536, ADO78564, AEB79485, AEB79550, AEK69633, AET43468, AF161810-AF161815, AF170831-AF170833, AF173179, AF173181-AF173208, AF173211 - AF173214, AF174300 - AF174302, AF260928, AF260929, AF284776-AF284778, AF469676 - AF469679, AF506016, AF541921, AFJ68184, AFJ68397, AFK27432, AJ236757 - AJ236770, AJ236772 - AJ236774, AJ236778 - AJ236782, AJ400634, AY159630 - AY159632, AY159634 - AY159639, AY159641, AY159643, AY159644 - AY159647, AY353727, AY353728, AY353730 - AY353739, AY353741 - AY353746, AY353753 - AY353765, AY353767 - AY353790, AY353792 - AY353805, AY601540 - AY601544, AY629562, BAD84188, BAF97950, CAB36938, D88830, DQ146647, DQ146658, DQ146669, DQ146680, DQ189233 - DQ189237, DQ189240, DQ299876, DQ339147 - DQ339151, DQ490543, DQ492678, DQ525182 - DQ525188, EF011980, EF033204, EF059918, EF059919, EF059924, EF159574, EU679378 - EU679380, GAU78558, Q9YJN7, U59108, U59110. 

## 4. Conclusions

The presence of intra-genotypic clusters and punctual amino acid variations in the NSP4 genotype E1 may indicate that NSP4 mutates mainly via accumulation of single point mutations. Since most of the variations in NSP4 fell in the carboxylic terminal region, especially in the VP4 binding site segment, it is important to consider that NSP4 is involved in morphogenesis and pathogenesis activities. Further analysis of NSP4 in the VP4 and VP6 binding site segment should be studied, especially with respect to structural conformational changes caused by amino acid variations. NSP4 is an important factor in rotavirus pathogenesis, and in this study an analysis examining the amino acid variations in the sequence and the gastroenteritis severity score was performed. The results failed to show a relationship between punctual variations in NSP4 and the severity of rotavirus gastroenteritis. The study of the NSP4 protein and its interaction with other viral proteins may aid our understanding of the pathogenesis of the rotavirus.
